# Field‐based experimental water footprint study of sunflower growth in a semi‐arid region of China

**DOI:** 10.1002/jsfa.7726

**Published:** 2016-04-20

**Authors:** Lijie Qin, Yinghua Jin, Peili Duan, Hongshi He

**Affiliations:** ^1^ School of Geographical SciencesNortheast Normal UniversityChangchun130024China; ^2^ School of Natural ResourcesUniversity of MissouriMO65211USA

**Keywords:** water footprint, blue water, green water, grey water, sunflower, semi‐arid region

## Abstract

**BACKGROUND:**

Field‐scale changes in the water footprint during crop growth play an important role in formulating sustainable water utilisation strategies. This study aimed to explore field‐scale variation in the water footprint of growing sunflowers in the western Jilin Province, China, during a 3‐year field experiment. The goals of this study were to (1) determine the components of the ‘blue’ and ‘green’ water footprints for sunflowers sown with water, and (2) analyse variations in water footprints and soil water balance under different combinations of temperature and precipitation. Specific actions could be adopted to maintain sustainable agricultural water utilisation in the semi‐arid region based on this study.

**RESULTS:**

The green, blue, and grey water footprints accounted for 93.7–94.7%, 0.4–0.5%, and 4.9–5.8%, respectively, of the water footprint of growing sunflowers. The green water footprint for effective precipitation during the growing season accounted for 58.8% in a normal drought year but 48.2% in an extreme drought year. When the effective precipitation during the growing season could not meet the green water use, a moisture deficit arose. This increase in the moisture deficit can have a significant impact on soil water balance.

**CONCLUSION:**

Green water was the primary water source for sunflower growth in the study area, where a scarcity of irrigation water during sunflower growth damaged the soil water balance, particularly in years with continuous drought. The combination of temperature and precipitation effected the growing environment, leading to differences in yield and water footprint. The field experiments in this area may benefit from further water footprint studies at the global, national and regional scale. © 2016 The Authors. *Journal of The Science of Food and Agriculture* published by John Wiley & Sons Ltd on behalf of Society of Chemical Industry.

## INTRODUCTION

Agriculture is the largest consumer of freshwater, accounting for more than 70% of the world's water use.^1^, ^2^ Water is an important resource for maintaining crop production, and ensuring water security for crop production has become a global problem. Therefore, the evaluation of water resource consumption during crop production is very important.

The introduction of the water footprint concept and its mathematical modelling have provided a new perspective on analysing water resource utilisation and management activities for crop production.^3^, ^4^, ^5^ The water footprint model reflects both water use and water source.^6^, ^7^, ^8^, ^9^, ^10^, ^11^ The water footprint for crop growth is defined as the volume of fresh water required for growth and for diluting pollutants during crop growth, and comprises blue, green and grey water footprints.^12^, ^13^, ^14^, ^15^, ^16^, ^17^ Many studies on the water footprint of crop growth have promoted the development of water footprint theory.^18^, ^19^, ^20^, ^21^, ^22^, ^23^, ^24^, ^25^, ^26^, ^27^

To understand the water sources utilised for crop growth, a distinction is made between blue and green water. The visible liquid water, blue water, consists of the surface and ground water formed from rainfall including rivers, lakes, and water contained in aquifers, which is typically viewed as irrigation water during crop growth. Green water is the rainfall that infiltrates into the unsaturated soil layer and is available for crop growth.^28^, ^29^, ^30^, ^31^ Green water is defined as the effective precipitation during the growing season. When a crop receives full irrigation, green water is calculated first and is equal to the effective precipitation during the growing season, and blue water is subsequently derived using green water, i.e. the volume of blue water equals the crop water use for growth minus green water,^13^, ^17^, ^21^, ^32^ which is designated as the ‘crop water requirement option’ (CWR option) in the CROPWAT model. Irrigation conditions in different regions may vary during the growing season and can range from full irrigation to inadequate or no irrigation. Thus, the ‘irrigation schedule option’ in the CROPWAT model should be applied, which allows the specification of the actual irrigation water application. In this option, blue water (irrigation water) is first defined, and then green water.^21^ In earlier studies of water footprints, the specific crop water use for growth was calculated assuming full irrigation because of the lack of data on the irrigated area and irrigation volume for specific crops. However, full irrigation is realised for only a few crops in some areas. Therefore, the ‘irrigation schedule option’ should be chosen and widely applied for all conditions. This study used the ‘irrigation schedule option’ to evaluate blue and green water footprints.

Applying fertiliser and pesticides can lead to water pollution. To prevent water pollution and ensure that water quality meets safety standards, fresh water is required to dilute various nutrient elements (such as N, P, K) and pesticides. The fresh water used for such a purpose, particularly for the dilution of leached nitrogen, is called grey water.^21^, ^33^ Leached nitrogen is estimated to account for 5–15% of the nitrogen in fertilisers.^34^, ^35^

The water footprint can be assessed at different spatiotemporal scales, e.g. global averages using annual data; national, regional or catchment‐specific averages using annual or monthly data; and small catchment or field‐specific averages using monthly or daily data.^21^ At present, studies on the water footprint of crop growth use a basic method of statistical analysis at the global, national or regional scales. Few, however, have focused on the water footprint of crop growth at a field‐specific scale.

Drought occurs on an almost annual basis in the western Jilin Province of north‐east China, and agricultural water shortages are severe. Sunflower is a major cash crop in this region, with the sown area accounting for >70% of the entire acreage of sunflower production in Jilin Province. Water is a major constraint on sunflower growth in this region. Because of severe spring droughts, the production technology of sunflowers involves sowing with water, in which the seed is irrigated with a small quantity of water at the time of sowing, creating a microenvironment with sufficient soil water to ensure germination and seedling establishment and having no irrigation water at any other stage. To compare the results of water utilisation efficiency, we conducted field experiments over a 3‐year period under identical soil conditions and farmland management to determine the characteristics of the sunflower water footprint on a field‐specific scale.

## STUDY AREA AND METHODS

### Study area

**Figure 1:**
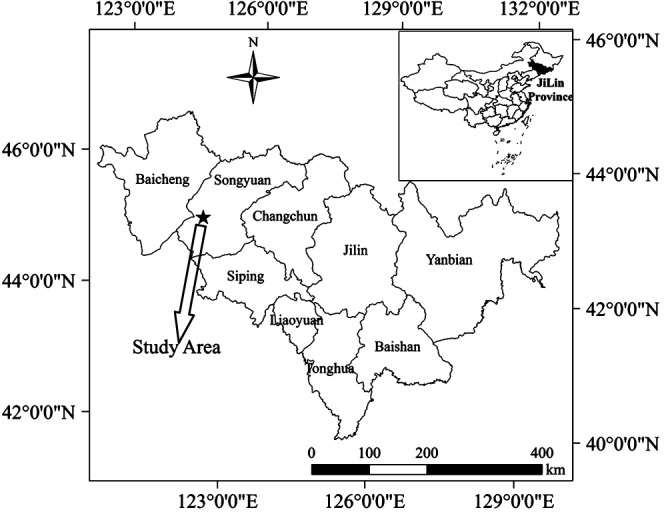
Location of the study area.

The study area was located at the Research Station of the Songnen Grassland Ecosystem, Northeast Normal University (Changling, Jilin Province, China, 44° 40′ N, 123° 44′ E) (Fig. 1). The soil type is aeolian sandy soil and is composed of 61% sand, 18% silt and 21% clay. The soil water content at field capacity is 24.3% by weight, and the permanent wilting point is 3.6%. The soil is relatively low in organic matter content, but abundant in total and available nitrogen.^36^ The mean annual temperature of the study area is 5.2 °C. The active accumulated temperature (≥10 °C) is 2920 °C, and the number of frost‐free days is between 140 and 160. The mean annual precipitation of the study area is 453 mm, with >70% occurring between June and August. The annual mean evaporation is 1600 mm, which is approximately 3.5 times the level of precipitation. The terrain of the study area is smooth, without surrounding rivers. The groundwater depth is approximately 0–3 m during the crop growing season.^37^ Because there is a strong water permeability in the aeration zone, the groundwater is easily polluted. The annual mean wind speed is 3.5 m s^−1^ with the highest wind speeds occurring during the spring. Spring (March to May) droughts and gales (with wind speeds reaching 17.2–20.7 m s^−1^) are typical in this semi‐arid region. Because of the shortage of water resources, agricultural production in the region uses sowing with water.

### Field experiments

Confection sunflower is usually planted in early June in the study area, primarily in 65 cm‐wide rows with a plant spacing of 150–180 cm. Therefore, our experiments were conducted using sunflowers in 65 cm‐wide rows with a plant spacing of 150 cm. The seeds were Sandaomei, which is the most common cultivar in the area. In each year, 20 m^3^ hm^−2^ of water was used on the day of sowing. In addition, 6.5 g of (NH_4_)_2_HPO_4_ (N–P–K, 18‐46‐0) per plant was applied uniformly at the bottom of each furrow. In late July, between the tilling treatments, plants were fertilised with 6.5 g of urea per plant, followed by the building of new ridges with a plough. The experiment included four replicates in 30 m long × 3.9 m wide plots. In each plot, all of the treatments were the same, including the sowing time and method, amount of fertiliser, and other farmland management activities.

**Figure 2:**
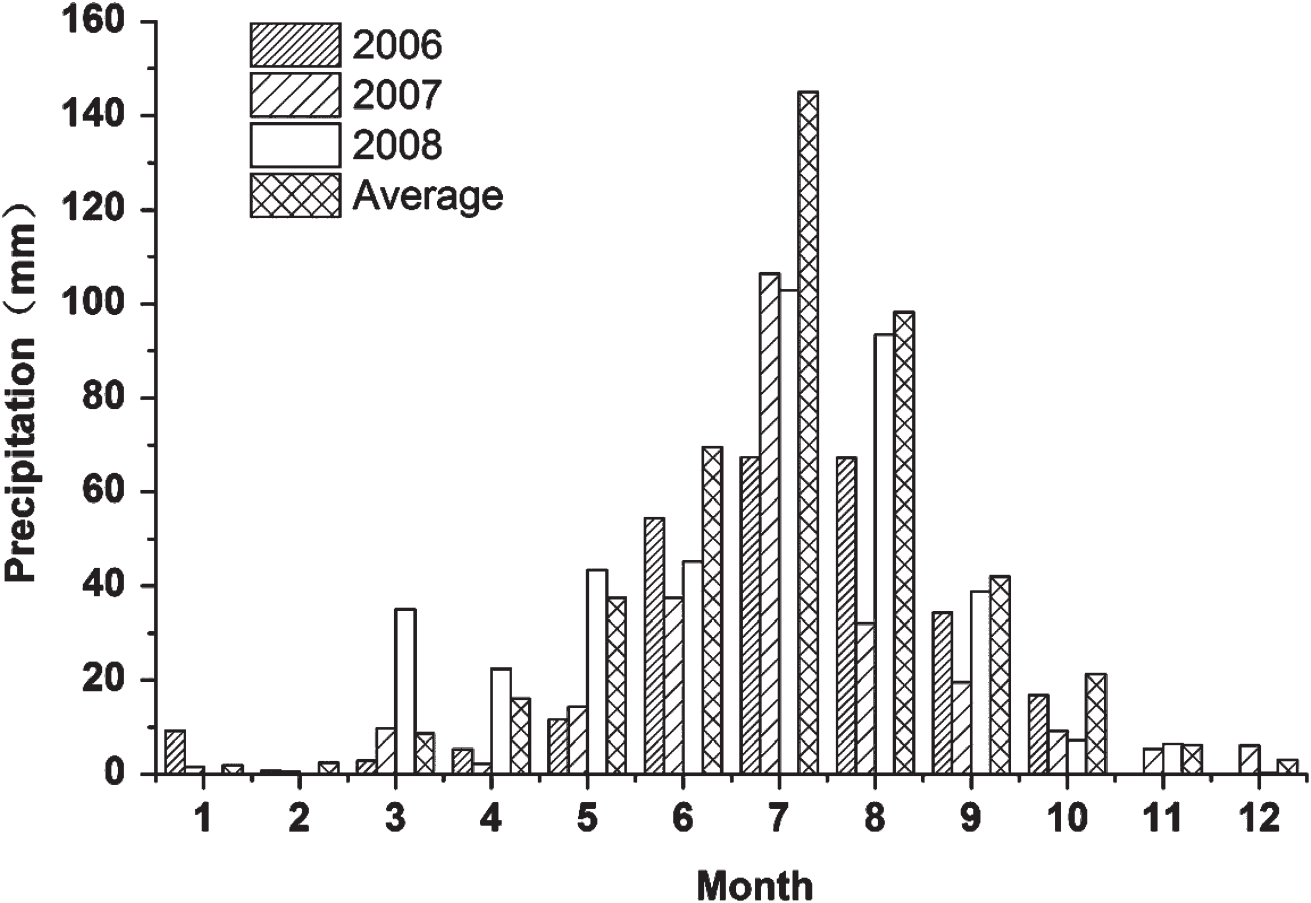
Monthly precipitation during each experimental year (2006–2008) and the 56‐year (1953–2008) average.

The experiments were conducted from 2006 to 2008. Seeds were sown on 5 June 2006, 25 May 2007, and 25 May 2008. The harvest date was the same (10 October) each year. The average precipitation in the study area during the sunflower growing season (June to September) from 1953 to 2008 was 349.0 mm. The total precipitation during the sunflower growing season was 223.4, 195.6 and 280.5 mm in 2006, 2007 and 2008, respectively. Thus, 2008 was characterised as a normal drought year, whereas 2006 was characterised as a severe drought year. Not only was the precipitation during the 2007 sunflower growing season low, but the temporal distribution was also uneven (Fig. 2); therefore, 2007 was considered an extreme drought year.

### Water footprint calculation

**Figure 3:**
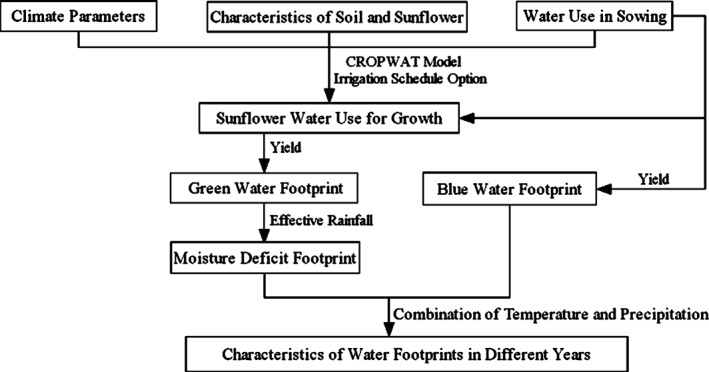
Diagram of water footprint assessment.

First, sunflower water use during the growing season was determined based on the climate parameters and irrigation water, calculated using the ‘irrigation schedule option’ in the CROPWAT model. Next, the water footprints of sunflower were calculated from water use and yield. Finally, the characteristics of the water footprints of sunflower were compared among the 3 years. The overall calculation process is shown as a flow chart in Fig. 3.

#### Sunflower water use

Sunflower water use during the growing season was equal to evapotranspiration, which was calculated using the Penman–Monteith model.

First, the reference evapo‐transpiration (ET_0_, mm) under the influence of climatic factors was calculated^38^, ^39^ as follows:

(1) $${ET}_{0}=\frac{0.408\Delta \left(R_{n}-G\right)+\gamma \frac{900}{T+273}U_{2}\left(e_{s}-e_{a}\right)}{\Delta +\gamma \left(1+0.34U_{2}\right)}$$

where R_n_ is the net radiation (MJ m^−2^ day^−1^), G is the soil heat flux (MJ m^−2^ d^−1^), T is the mean temperature (°C), U_2_ is the wind speed at 2 m height (m s^−1^), e_s_ is the saturation vapour pressure (kPa), e_a_ is the actual vapour pressure (kPa), Δ is the slope of saturation vapour pressure versus the air temperature curve (kPa °C^−1^), and γ is the hygrometer constant (kPa °C^−1^).

The reference evapo‐transpiration (ET_0_) was then multiplied by the water stress coefficient (K_s_) and the sunflower parameter (K_c_)^40^ to calculate the evapo‐transpiration of sunflower (ET_a_, mm):

(2) $${ET}_{a}=K_{s}\times K_{c}\times \;{ET}_{0}$$

The ET_a_ was then converted into the volume of sunflower water use (SWU, m^3^ hm^−2^):

(3) $$SWU=10\;\times \;{ET}_{a}$$

such that the factor of 10 converts water depth (mm) into water volume per area (m^3^ hm^−2^).

#### Blue water footprint

The sunflower production mode was to sow with water; thus, the volume of blue water comprised only the water use on the sowing day. The formula used for the blue water footprint of sunflower was:

(4) $${WF}_{\text{blue}}=\frac{\mathrm{IU}}{Y}$$

where WF_blue_ is the blue water footprint of sunflower (m^3^ kg^−1^), IU is the water use on the sowing day (m^3^ hm^−2^), and Y is the sunflower yield (kg hm^−2^).

#### Green water footprint

The green water was defined as the green water use equivalent to the sunflower water use for growth minus the water use on the sowing day. The formula used for the green water footprint of sunflower was:

(5) $${WF}_{\text{green}}=\frac{GWU}{Y}=\frac{SWU-\mathrm{IU}}{Y}$$

where WF_green_ is the green water footprint of sunflower (m^3^ kg^−1^), and GWU is the green water use (m^3^ hm^−2^).

Typically, green water use is equivalent to the effective precipitation during the crop growing season. However, crop production under inadequate irrigation or no irrigation makes the effective precipitation insufficient for water use; thus, the difference, called the moisture deficit, is needed to supply the remainder of the water use in crop production, which may come from the effective precipitation during the non‐growing season or the condensation of atmospheric water.

Because drought conditions are common in the study area, the soil water balance was not maintained during sunflower growth. When the effective precipitation during the growing season could not meet the green water use, i.e. EP < SWU‐IU, the soil water was not balanced, and there was a moisture deficit.

In this case, the formula used for the moisture deficit was:

(6) MD=SWU−IU−EP

where MD is the moisture deficit during the growing season (m^3^ hm^−2^) and EP is the effective precipitation during the growing season (m^3^ hm^−2^).

The effective precipitation (P_eff_, mm) is calculated according to the method developed by the US Department of Agriculture (USDA), as follows:

(7) $$P_{\text{eff}}=\left\{\begin{array}{c} P_{\text{total}}\left(4.17-0.2P_{\text{total}}\right)/4.17\\ 4.17+0.1P_{\text{total}} \end{array}\right.\begin{array}{c} P_{\text{total}}<8.3\;\mathrm{mm}\\ P_{\text{total}}\geq 8.3\;\mathrm{mm} \end{array}$$

(8) $$EP=10\;\times P_{\text{eff}}$$

where P_eff_ is the effective precipitation (mm) and P_total_ is the total precipitation (mm), both at a daily time step. The factor of 10 converts water depth (mm) into water volume per area (m^3^ hm^−2^).

From this, Eqn (5) can be further defined as follows:

(9) $${WF}_{\text{green}}={WF}_{EP}+{WF}_{MD}=\frac{EP}{Y}+\frac{MD}{Y}$$

where WF_EP_ is the water footprint of the effective precipitation (m^3^ kg^−1^), and WF_MD_ is the water footprint of the moisture deficit (m^3^ kg^−1^).

#### Grey water footprint

In this study, 10% of the nitrogen fertiliser was assumed to represent the amount of leached nitrogen. The terrain of the study area is smooth, without surrounding rivers, and the leached nitrogen infiltrates the soil, potentially causing groundwater pollution. Therefore, it was necessary to calculate the amount of dilution water required to meet the nitrogen standard for groundwater. We used grade III of the groundwater quality standard of China (GB/T 14848–93), in which the nitrogen in the groundwater cannot exceed 20 mg L^−1^.

The grey water footprint of sunflower was calculated as:

(10) $${WF}_{\text{grey}}=\frac{\left(\alpha \times AR\right)/\left(c_{\max}-c_{nat}\right)}{Y}$$

where WF_grey_ is the grey water footprint of sunflower (m^3^ kg^−1^), AR is the quantity of nitrogen fertiliser applied (kg hm^−2^), α is the leaching‐run‐off fraction of nitrogen fertiliser, C_max_ is the nitrogen standard in groundwater (kg m^−3^), and C_nat_ is the natural concentration of nitrogen (kg m^−3^).

#### Water footprint of sunflower growth

The water footprint of sunflower growth was calculated as the total water consumption during the sunflower growing season, including the blue, green and grey water footprints:

(11) $${WF}_{\text{prod}}=\;{WF}_{\text{blue}}+\;{WF}_{\text{green}}+\;{WF}_{\text{grey}}$$

where WF_prod_ is the water footprint of growing sunflowers (m^3^ kg^−1^).

## RESULTS

### Water use for sunflower growth in different years

**Figure 4:**
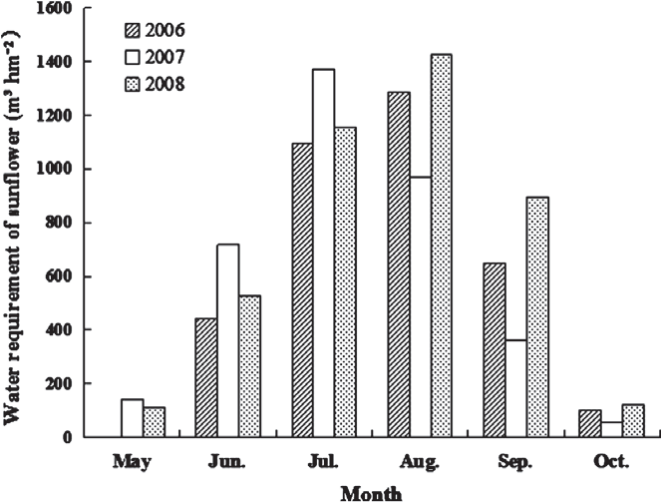
Water use during the sunflower growing season in 2006, 2007 and 2008.

In each year, the sunflower water use increased steadily to a peak period between July and August; water use subsequently decreased (Fig. 4). At the initial stage of growth, the sunflower plants were small, and the water use was low. As the plants grew during July, transpiration increased with increasing leaf area; therefore, sunflower water use increased and reached a maximum in July or August.

The precipitation in 2008 was the highest among the 3 years, and the total sunflower water use in 2008 was also the highest. The proportion of sunflower water use during the peak period relative to the total sunflower water use in 2008 was 60.9%, the lowest among the 3 years. The total sunflower water use during the growing season in 2006 was the lowest among the 3 years, whereas the proportion of sunflower water use during the peak period relative to the total sunflower water use in 2006 was 66.6%, the highest among the 3 years. In the extreme drought year of 2007, the precipitation was low and uneven. Hence, the total sunflower water use in 2007 was intermediate, and the proportion of sunflower water use during the peak period relative to the total sunflower water use was 64.8%, slightly lower than in 2006 and intermediate between 2006 and 2008.

### Blue and green water footprints of sunflower growth

In the study area, the sunflower water use from blue water was the water used on the sowing day. All other water uses were derived from green water. Although the treatments (including experimental field, seeds, sowing method, and field management) were the same during the 3 years, the sunflower yields were different due to variation in the climatic conditions; thus, the blue and green water footprints of the sunflower were also different, particularly the green water footprint (Table 1).

**Table 1:** Blue and green water footprints of sunflower in 2006, 2007 and 2008

Year	SWU (m^3^ hm^−2^)	IU (m^3^ hm^−2^)	GWU (m^3^ hm^−2^)	Y (kg hm^−2^)	Blue WF (m^3^ kg^−1^)	Green WF (m^3^ kg^−1^)
2006	3571	20	3551	2790	0.007	1.273
2007	3613	20	3593	2145	0.009	1.675
2008	4240	20	4220	2456	0.008	1.718

SWU represents the volume of sunflower water use; IU represents water use on the sowing day; GWU represents the green water use; Y represents the sunflower yield; Blue WF represents the blue water footprint; Green WF represents the green water footprint.

The results showed that the blue water footprint of sunflower was 0.007–0.009 m^3^ kg^−1^, which indicated that the proportion of the blue water footprint relative to water use per unit mass was <1%. Conversely, the green water footprint of sunflower was 1.273–1.718 m^3^ kg^−1^, which indicated that the proportion of the green water footprint relative to water use per unit mass was >99%. Overall, the green water footprint of sunflower in 2008 was the highest among the 3 years, whereas in 2006, it was the lowest.

**Figure 5:**
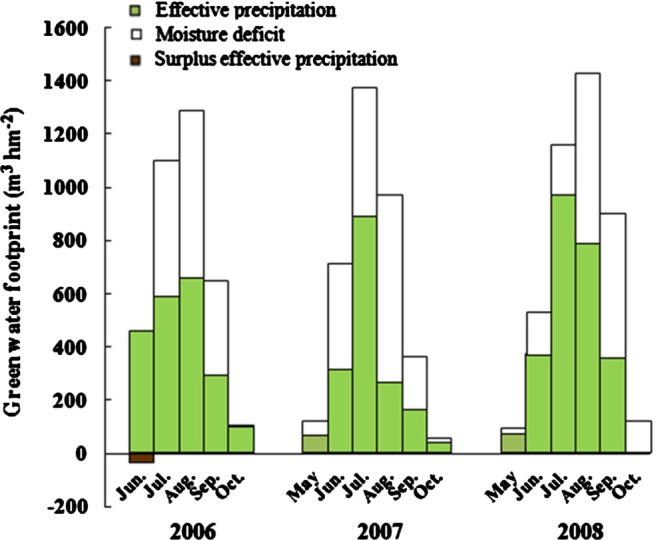
Comparison of green water footprints for sunflower in 2006, 2007 and 2008.

Normally, drought prevails almost annually in the study area, and the annual and inter‐annual precipitations are both all uneven. On most days during the 3‐year period, the effective precipitation did not meet the sunflower water use (Fig. 5). Thus, the sunflower green water use comprised two parts: the effective precipitation and the moisture deficit.

The green water footprints differed among the 3 years (Table 2). In 2006, the sunflower seeds were sown in early June. In addition to the water use on the sowing day, the effective precipitation in June could more than satisfy the sunflower water use. The surplus of effective precipitation in June was 36 m^3^ hm^−2^, which reduced the moisture deficit in July. There was no subsequent surplus of effective precipitation in any other month during the growing season. Overall, the volume of effective precipitation in 2006 was 2088 m^3^ hm^−2^, and the moisture deficit was 1463 m^3^ hm^−2^. The water footprint of effective precipitation in 2006 was 0.748 m^3^ kg^−1^ (58.8% of the green water footprint), and the footprint of the moisture deficit was 0.524 m^3^ kg^−1^ (41.2% of the green water footprint). In 2007, the effective precipitation during the growing season could not satisfy the sunflower water use in any month. The volume of effective precipitation in 2007 was 1732 m^3^ hm^−2^, and the moisture deficit was 1861 m^3^ hm^−2^. The water footprint of effective precipitation in 2007 was 0.807 m^3^ kg^−1^ (48.2% of the green water footprint), and the footprint of the moisture deficit was 0.868 m^3^ kg^−1^ (51.8% of the green water footprint). In 2008, the effective precipitation during the growing season could not satisfy the sunflower water use in any month. The volume of effective precipitation in 2008 was 2501 m^3^ hm^−2^, and the moisture deficit was 1719 m^3^ hm^−2^. The water footprint of effective precipitation in 2008 was 1.018 m^3^ kg^−1^ (59.3% of the green water footprint), and the footprint of the moisture deficit was 0.700 m^3^ kg^−1^ (40.7% of the green water footprint).

**Table 2:** Green water footprint of sunflower in 2006, 2007 and 2008 (m^3^ kg^−1^)

Year	May		June		July		August		September		October	
	WF_EP_	WF_MD_	WF_EP_	WF_MD_	WF_EP_	WF_MD_	WF_EP_	WF_MD_	WF_EP_	WF_MD_	WF_EP_	WF_MD_
2006	–	–	0.158	0	0.215	0.170	0.235	0.225	0.105	0.127	0.035	0.002
2007	0.025	0.030	0.149	0.185	0.415	0.225	0.124	0.328	0.076	0.092	0.018	0.008
2008	0.008	0.029	0.151	0.064	0.395	0.075	0.319	0.262	0.145	0.220	0	0.050

WF_EP_ represents the water footprint of effective precipitation; WF_MD_ represents the water footprint of the moisture deficit.

### Grey water footprint of sunflower

The amount of fertiliser applied during sunflower growth was 134 kg hm^−2^, which consisted of 67 kg hm^−2^ of (NH_4_)_2_HPO_4_ and 67 kg hm^−2^ of urea in each of the 3 years. Based on the nitrogen content of (NH_4_)_2_HPO_4_ and urea, the amount of leached nitrogen was 4.34 kg hm^−2^ each year. Leached nitrogen infiltrates the groundwater in the study area. To ensure that the nitrogen concentration of the groundwater met grade III of the groundwater quality standard of China (GB/T 14848–93), freshwater was needed to dilute the fertiliser. Thus, the grey water footprints of sunflower were 0.078 m^3^ kg^−1^, 0.101 m^3^ kg^−1^, and 0.088 m^3^ kg^−1^ in 2006, 2007 and 2008, respectively, based on Eqn (10). The level of fertilisation used in this experiment was low compared with that used in local crop production. Generally, the grey water footprint increased as more fertiliser was used.

### Water footprint of sunflower growth

The results showed that changes in the water footprint of sunflower growth were closely related to the climatic conditions. During the serious drought year of 2006, the rainfall distribution pattern was normal, and the water footprint of sunflower growth was 1.358 m^3^ kg^−1^, in which the blue, green, and grey water footprints accounted for 0.5%, 93.7% and 5.8%, respectively. In the extreme drought year of 2007, the precipitation was not only low but also uneven; the water footprint of sunflower growth was 1.785 m^3^ kg^−1^ and comprised 0.5% blue water footprint, 93.8% green water footprint, and 5.7% grey water footprint. In the normal drought year of 2008, the water footprint of sunflower growth was 1.814 m^3^ kg^−1^, consisting of 0.4% blue water footprint, 94.7% green water footprint, and 4.9% grey water footprint. These results also showed that the green water footprint was the primary part of the water footprint of sunflower growth, accounting for more than 93% of the water footprint.

## DISCUSSION

Spring drought is the primary type of drought in the semi‐arid region of northern China and causes soil moisture deficiency and a low rate of seedling emergence. As a result, the sowing date must be postponed, and the growth period of the crop is shortened, which significantly affects crop yield. Sowing with water is a proven, effective measure that addresses the challenge of spring drought and ensures seedling emergence.^41^

This study explored the water footprint of sunflower based on the actual irrigation water used for sunflower growth. In the study area, it is difficult to achieve full irrigation because of limited water resources. To achieve high yield and combat the severe spring drought, the production technology of sowing with water was applied, which uses only a small amount of water on the sowing day and not full irrigation. For this reason, the ‘irrigation schedule option’ in the CROPWAT model was more accurate than the ‘CWR option’ in the calculation of the sunflower water footprint for the study area. A comparison of the results obtained from these two options revealed noticeable differences (Table 3), in which the volume of water use for sunflower from the ‘CWR option’ was larger than from the ‘irrigation schedule option’, the blue water footprint was much greater than from the ‘irrigation schedule option’, and the green water footprint was lower than from the ‘irrigation schedule option’. It was necessary to distinguish between blue and green water footprints under the conditions of actual regional agricultural water use. The results from the ‘CWR option’ could not reflect the actual situation of local agricultural production with respect to water consumption. In this study, the soil water was not balanced during the growing season, and there was a moisture deficit because the effective precipitation during the growing season could not account for all the green water use, which is consistent with examples under the rain‐fed and irrigation scenarios using the irrigation schedule option in the water footprint assessment manual.^21^ The discussion in this study provides a basis for further studies on the water footprint of crop growth in a semi‐arid region. Furthermore, it reinforces the importance of green water in agricultural production^42,43^ and strengthens the case for soil water balance monitoring in a semi‐arid region.

**Table 3:** Comparison of water footprint results calculated using the ‘CWR option’ and the ‘irrigation schedule option’

Year	CWR option			Irrigation schedule option		
	Water use (m^3^ hm^−2^)	Blue WF (m^3^ kg^−1^)	Green WF (m^3^ kg^−1^)	Water use (m^3^ hm^−2^)	Blue WF (m^3^ kg^−1^)	Green WF (m^3^ kg^−1^)
2006	4246	0.773	0.748	3571	0.007	1.273
2007	5306	1.666	0.807	3613	0.009	1.675
2008	4616	0.861	1.018	4240	0.008	1.718

Water use represents the volume of sunflower water use during the sunflower growing season; Blue WF represents the blue water footprint; Green WF represents the green water footprint.

**Figure 6:**
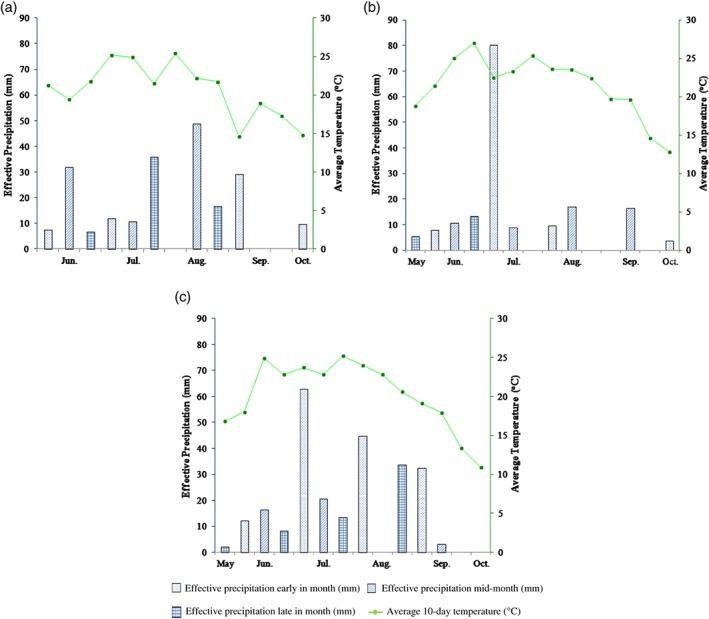
Comparison of dekadal temperature and effective precipitation during the sunflower growing season in 2006 (a), 2007 (b), and 2008 (c).

The variation in meteorological parameters was the major constraint on the value of the sunflower water footprint during the 3‐year field experiment because the production conditions, e.g. soil conditions, sowing date and method, fertiliser amount and farmland management, were the same in all years. There was no irrigation water except the sowing water used in the study area; effective precipitation was the primary limiting factor. However, the field experimental results showed that yield may not be higher, or water footprint, lower in a year with higher effective precipitation during the growing season. Therefore, the combination of temperature and precipitation was crucial to sunflower growth, and annual variation in the combination of temperature and precipitation produced a significantly different sunflower growing environment. A comparison of the dekadal effective precipitation and average temperature during the sunflower growing season in 2006, 2007 and 2008 is shown in Fig. 6.

In 2006, the combination of temperature and precipitation was well suited to the demands of sunflower growth. There was both precipitation and high temperature during the critical growing period for sunflower, which was advantageous for budding, flowering and maturation; therefore, the yield was the highest, and the water footprint was the lowest in 2006 among the 3 years. In 2007, the temperature was high, but the precipitation was low and uneven. The effective precipitation in May and June was very low, with only 21.6% of the effective precipitation for the growing season, which was not beneficial for seedlings. Subsequently, the effective precipitation during July was relatively adequate, providing good conditions for the growth and development of sunflower. In contrast, the effective precipitation during August was too low, with a significant negative effect on flowering and maturation stages. The high temperature and low precipitation at the early and later stages was not favourable for sunflower growth and development, so the yield in 2007 was the lowest among the 3 years, and the water footprint was moderate and 31.6% higher than that in 2006. In 2008, the precipitation was the highest among the 3 years, but the combination of temperature and precipitation in 2008 was not better than in 2006. The effective precipitation during June in 2008 was less than in 2006, making the seedling growth conditions in 2008 inferior to those in 2006. There was no effective precipitation during mid‐August in 2008, which was not beneficial for flowering and maturation. Therefore, the yield in 2008 was intermediate, lower than in 2006 and higher than in 2007. However, the water use in 2008 was the greatest among the 3 years, which led to the highest water footprint. Thus, the combination of temperature and precipitation affected the production environment and led to differences in yield and water footprint.

Studies on the water footprint of crop growth provide a basis for analysing global water footprint differences and national or regional water footprint characteristics that are based on statistical analyses. However, statistics reflect only the average value of an index, not the spatial differences within a region, and there is a lack of data on the irrigated area and irrigation volume for specific crops. Thus, further study of the water footprint is limited by the available data. Field experiments should be conducted to obtain data on crop variety, sowing date, fertiliser amount, water use and yield to accurately reflect local crop growth and supplement these data deficiencies. Field experiments can explore the variation in the water footprint of crop growth under the same soil conditions and farmland management. Furthermore, field studies can consider the sources of the crop water use and its impact on the environment, and several typical field experiments within a given area may benefit regional‐scale studies on the water footprint of crop growth, which may provide a basis for further water footprint studies at the global or national scale. Therefore, field experiments should be emphasised in water footprint studies, and long‐term field experiments are the most desirable to capture trends in the water footprints. In addition, the complexity of field experiments at a larger scale should be considered; the categorisation of typical regions in the experimental area would reflect the regional features and differences, which would be conducive to understanding the complexity. The typical regions should be divided with regards to meteorological conditions, soil and farmland management, and the differences in crop water footprint in these typical regions should reflect the characteristics of agricultural water utilisation in the experimental area.

## CONCLUSIONS

Within the study area, blue water was the water used on the sowing day, and green water consisted of the effective precipitation and moisture deficit. Green water was the primary water source for sunflower growth and accounted for 93.7–94.7% of the water footprint of sunflower growth. The blue water footprint accounted for 0.4–0.5% of the water footprint of sunflower growth, and the grey water footprint was between 4.9% and 5.8% of the water footprint. The water footprint of effective precipitation accounted for 58.8% of the green water footprint in the normal drought year, whereas in the extreme drought year, the water footprint of effective precipitation accounted for 48.2% of the green water footprint, which had a serious impact on soil water balance.

Green water occupies an important position in agriculture production for a semi‐arid region. The key way to ensure the sustainable development of agriculture is to protect the local green water resources. In this study, when the effective precipitation during the growing season could not meet the green water use, an increased moisture deficit occurred, which increased the impact on soil water balance. Therefore, if crop production without irrigation continues to be applied in the study area, the soil moisture content will gradually decrease in continuous drought years, which will damage the soil water balance and ultimately affect the sustainable development of agriculture in the region. To maintain the soil water balance, the appropriate irrigation scheme should be adopted in continuous drought years.
